# Opposing function of MYBBP1A in proliferation and migration of head and neck squamous cell carcinoma cells

**DOI:** 10.1186/1471-2407-12-72

**Published:** 2012-02-17

**Authors:** Gustavo A Acuña Sanhueza, Leonie Faller, Babitha George, Jennifer Koffler, Vinko Misetic, Christa Flechtenmacher, Gerhard Dyckhoff, Peter P Plinkert, Peter Angel, Christian Simon, Jochen Hess

**Affiliations:** 1 Department of Otolaryngology, Head and Neck Surgery, University Hospital Heidelberg, 69120 Heidelberg, Germany; 2 Junior Research Group Molecular Mechanisms of Head and Neck Tumors, German Cancer Research Center (DKFZ), DKFZ-ZMBH Alliance, 69120 Heidelberg, Germany; 3 Division of Signal Transduction and Growth Control, German Cancer Research Center (DKFZ), DKFZ-ZMBH Alliance, 69120 Heidelberg, Germany; 4 Institute of Pathology, University Hospital Heidelberg, 69120 Heidelberg, Germany

## Abstract

**Background:**

Head and neck squamous cell carcinoma (HNSCC) is one of the most prevalent and lethal cancers worldwide and mortality mostly results from loco-regional recurrence and metastasis. Despite its significance, our knowledge on molecular, cellular and environmental mechanisms that drive disease pathogenesis remains largely elusive, and there are limited therapeutic options, with only negligible clinical benefit.

**Methods:**

We applied global gene expression profiling with samples derived from a recently established mouse model for oral cancer recurrence and identified a list of genes with differential expression between primary and recurrent tumors.

**Results:**

One differentially expressed gene codes for Myb-binding protein 1a (MYBBP1A), which is known as a transcriptional co-regulator that physically interacts with nuclear transcription factors, such as NFκB and p53. We confirmed significantly reduced MYBBP1A protein levels on tissue sections of recurrent mouse tumors compared to primary tumors by immunohistochemistry, and found aberrant MYBBP1A protein levels also in tumor samples of HNSCC patients. Interestingly, silencing of MYBBP1A expression in murine SCC7 and in human HNSCC cell lines elicited increased migration but decreased cell growth.

**Conclusion:**

We provide experimental evidence that MYBBP1A is an important molecular switch in the regulation of tumor cell proliferation versus migration in HNSCC and it will be a major challenge for the future to proof the concept whether regulation MYBBP1A expression and/or function could serve as a novel option for anti-cancer therapy.

## Background

Head and neck squamous cell carcinoma (HNSCC) is the most common type of head and neck cancer primarily affecting the mucosa of the upper aero digestive tract [1]. Tobacco and alcohol consumption are the main risk factors for HNSCC and have a multiplicative combined effect [1]. In recent years, there has been an increase in the annual incidence of HPV-related HNSCC, suggesting that a subset of HNSCC is a sexual transmitted disease with distinct pathogenesis and clinical features [2].

In spite of considerable advances in diagnosis, treatment and our understanding of the molecular alterations that occur in the pathogenesis of HNSCC, a high rate of local recurrences and distant metastasis aggravates the clinical situation, and the 5-year survival rate of patients with an advanced disease has remained at approximately 50% [3,4]. Therefore, HNSCC is still a treatment challenge and better biomarkers are desperately needed to identify patients at high risk for treatment failure and to define novel molecular drug targets for more effective and less toxic treatment modalities for patients with advanced tumors [4-6].

The development and use of animal models that closely resembles the pathogenesis and the histopathology of HNSCC in humans is essential to expand our knowledge on the underlying molecular mechanisms and to develop and improve novel strategies for translational anti-cancer research [7]. Recently, we established an orthotopic floor-of-mouth squamous cell carcinoma model in mice, in which local recurrences occurred at a high frequency [8]. In the current study, we applied global gene expression profiling with samples of this mouse model to identify genes with differential expression between primary and recurrent tumors. One candidate gene encoded the transcriptional co-regulator MYBBP1A. MYBBP1A was originally identified as an interacting partner of c-Myb, a proto-oncogene product [9]. It is localized mainly in the nucleoli [10] and during the last years was shown to bind several transcription factors, such as NFκB [11].

Here we provide experimental evidence for a crucial role of MYBBP1A as an important regulator of tumor cell proliferation and migration, and that tumor cells with reduced MYBBP1A expression may represent a sub-population of slow-cycling but mobile cells implicated in local tumor recurrence and metastasis.

## Methods

### Animal work and sample preparation

Primary and recurrent mouse tumors were obtained as described previously [8]. The procedures for performing animal experiments were in accordance with the principles and guidelines of the 'Arbeitsgemeinschaft der Tierschutzbeauftragten in Baden-Württemberg'and were approved by the 'Regierungspräsidium Karlsruhe', Germany (35-9185.81/G-21/07). Tumor samples for RNA preparation were immediately frozen in liquid nitrogen after isolation. For histological analysis, primary and recurrent tumors samples were embedded in OCT media (Tissue-Tek, Netherlands) or paraffin and subsequently cut in 6 μm sections.

### RNA preparation

Total RNA extraction from mouse tumors and human HNSCC cell lines was performed according to the manufacturer's instructions using peqGOLD RNAPure™ Reagent (Peq Lab, Erlangen, Germany). Total RNA concentration was determined by Nanodrop (Thermo Scientific, USA).

### Global gene expression profiling

Total RNA (1 μg) of primary and recurrent tumor samples derived from five animals were submitted to the Genomics Core Facilities of DKFZ (Heidelberg, Germany) for global gene expression profiling based on Illumina technology using whole genome BeadChip Sentrix arrays (mouse WG-6 v2). Array data GEO accession number: GSE35377. Data normalization and analysis was done as described in Supplemental data.

### IHC analysis

Paraffin-embedded tissue specimens of HNSCC patients were provided by the tissue bank of the National Center for Tumor Disease (Institute of Pathology, University Hospital Heidelberg) after approval by the local institutional review board). Paraffin-embedded mouse tumor samples were obtained as described before [8]. IHC staining was performed with the Immunodetection kit (Vector laboratories, Burlingame, CA) according to manufacturer's instructions and as described elsewhere [12]. Following antibodies were used: Anti-mouse MYBBP1A (AV37224, Sigma-Aldrich, Germany), anti-mouse Ki67 (Novacastra), anti-human MYBBP1A (ab54160, Abcam, UK).

### Cell culture

Human HNSCC cell lines were purchased from ATCC. Cells were maintained in Dulbecco's (SCC-4, SCC-9, SCC-25) or Minimum Essential Medium (Fadu, Cal-27) supplemented with 10% fetal bovine serum (Invitrogen, Germany), 2 mM L-Glutamine (Invitrogen, Germany) and Antibiotics (50 μg/ml Penicillin-Streptomycin, Invitrogen, Germany) in a humidified atmosphere of 6% CO_2 _at 37°C. The SCC-7 cell line was cultured as described previously [8].

### siRNA transfection

Human MYBBP1A-specific RNA-oligos (CAA AGG AGG UCA UAA GAA AUU) and oligos with scrambled sequence (GUC GAA UGC GAU UGU ACC GUU) were purchased from Sigma-Aldrich. Mouse siRNA was purchased from Santa Cruz Biotechnologies (sc-149729). The transfection in Cal-27 and in SCC-7 cell lines was performed using oligofectamine (Invitrogen, Germany) according to manufacturer's instructions. Analysis of human or mouse MYBBP1A expression silencing and all functional experiments were done 48 hours after transfection.

### Real-Time quantitative polymerase chain reaction

Real-time quantitative polymerase chain reaction (RQ-PCR) analysis was performed as described previously [13]. Primers used for RQ-PCR are listed in Additional file 1: Table S1. Target gene cycle of threshold values were normalized to the corresponding cycle of threshold of Hprt (for mouse samples) or LAMIN B (for human samples) using the change in cycle of threshold method.

### Western blot analysis

Isolation of nuclear extracts and Western blot analysis were performed as described previously [14]. Anti-human MYBBP1A (ab54160) and HSC70 antibodies (ab1427) were purchased from Abcam (UK). Anti-mouse Mybbp1a (sc-133880), anti-E-Cadherin (sc-8426), anti-Actin (SC-1615) and anti-Histone H1 (sc-8030) were purchased from Santa Cruz Biotechnology.

### Cell migration assay

Cells were plated to reach confluence one day prior the assessment of the experiment in the migration chambers (Culture inserts, Ibidi, Germany). Next day, the cells were treated with 10 μg ml^-1 ^of Mitomycin C (Sigma-Aldrich, Germany) for 30 minutes and washed twice with culture media. Once the chamber was removed pictures were taken at the time points 18, 24, 36 and 42 hours, and analyzed using the UTHSCSA Image tool.

### Cell growth and proliferation assay

Cell growth was assessed by Trypan-blue staining (Sigma-Aldrich, Germany) and counting the cell number under the microscope using a Neubauer chamber (Labor Optik, Germany). Proliferation of Cal-27 and SCC-7 was analyzed using the BrdU Flow Kit (BD Pharmigen, Germany) according the manufacturer's instructions. FACS analysis was performed using CellQuest Pro (BD Biosciences, Heidelberg, Germany). according to the manufacturer's instructions.

### Statistical analysis

Data are expressed as means ± SD of three independent experiments, each performed in triplicate. Differences between groups were assessed by unpaired, two-tailed Student's *t *test; P < 0.05 was considered significant (Chi^2 ^Test).

## Results

### Identification of differentially expressed genes in a mouse model of local oral cancer recurrence

**Figure 1:**
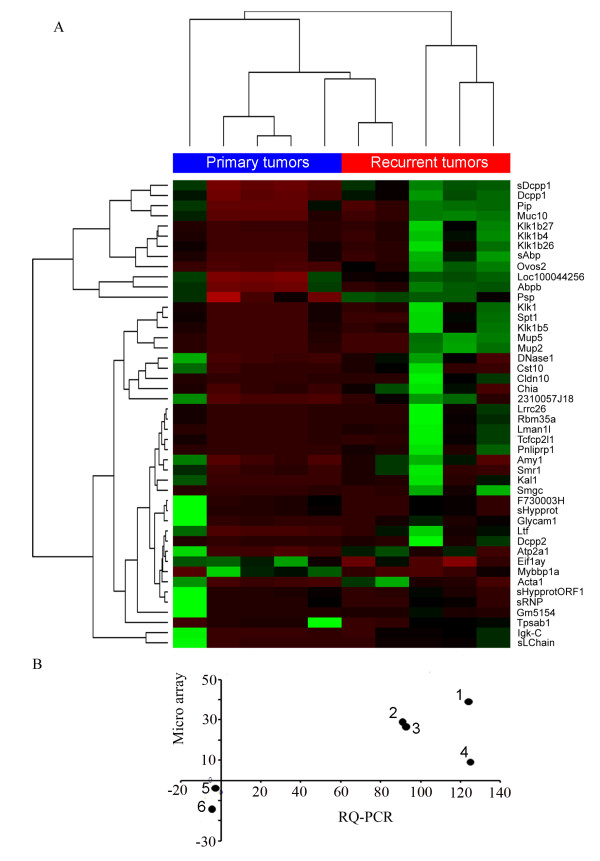
**Differentially expressed genes in recurrent tumors of a mouse model**. (**A**) Heat map of differentially expressed genes between primary and recurrent tumors of oral squamous cells carcinomas developed in a mouse model. (**B**) Altered transcript levels between primary tumors and local recurrences were confirmed for selected genes by quantitative RT-PCR (1 = Ovos2; 2 = Muc10; 3 = Dccp1; 4 = Smra3; 5 = Tpsa1a; 6 = Mybbp1a). Data for relative transcript levels of selected genes obtained from RQ-PCR analysis and global gene expression profiling were plotted.

To identify differentially expressed genes, whose expression might be involved in the formation and maintenance of local recurrence in oral squamous cell carcinoma, we applied global gene expression profiling on primary and recurrent tumor samples of an established orthotopic floor-of-mouth squamous cell carcinoma mouse model [8]. As described previously, primary tumors were generated by injection of murine SCC-7 cells into the floor-of-mouth of BALB/c-nu mice. Local recurrences were obtained after microsurgical removal of primary tumors. Total RNA was isolated from five pairs of primary tumor and respective local recurrence and applied to global gene expression analysis using whole genome arrays. Following data normalization we ranked differentially expressed genes according to their relative transcript levels in primary tumors and local recurrences of each individual and selected those genes with altered expression in at least three out of five tumor pairs for further analysis (Figure 1A). We identified a group of 49 differentially expressed gene IDs, of which 43 showed enhanced expression and 6 were down-regulated in local recurrences compared to primary tumors. We selected several genes (e.g. Ovos2, Smra3, Muc10, Dccp1, Tpsab1, and Mybbp1A) for quantitative RT-PCR analysis and could confirm altered transcript levels between primary tumors and local recurrences (Figure 1B).

### MYBBP1A protein expression in primary tumors, local recurrences and its correlation with proliferation and migration

**Figure 2:**
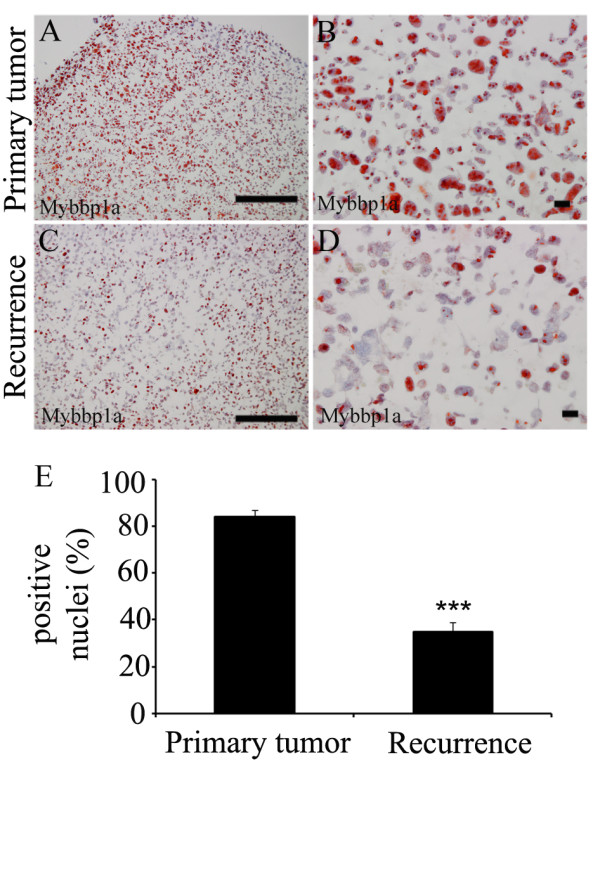
**MYBBP1A is down-regulated in recurrences of squamous cell carcinoma**. (**A-D**) Immunohistochemical analysis of mouse tumor sections with an anti-Mybbp1a antibody revealed specific staining (red signal) in nuclei of tumor cells. Counterstaining was done using hematoxylin. Scale bars, 50 μm. (**E**) Positive stained nuclei were quantified and plotted as percentage total (*** p-value = 0.0001).

**Figure 3:**
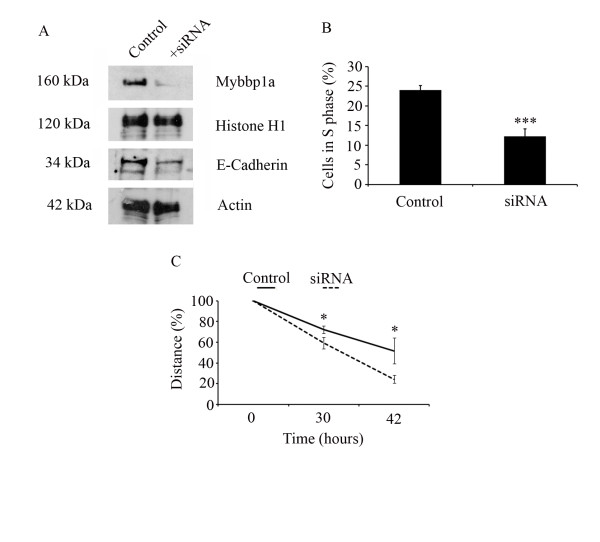
**Function of Mybbp1a on cell proliferation and migration in murine SCC-7 cells**. (**A**) Murine SCC-7 cells were transfected with scramble (control) or Mybbp1a-specific siRNA (+siRNA), and reduced expression of Mybbp1a protein was confirmed in nuclear fraction using a murine anti-Mybbp1a antibody. Histone H1 levels served as internal control of protein lysate quantity and quality. Silencing of Mybbp1a was accompanied by a reduction of E-Cadherin protein levels and an Actin control reveals equal whole cell lysate loading. (**B**) Transient Mybbp1a down-regulation led to a reduction of DNA synthesis quantified by BrdU incorporation (*** p-value = 0.0011). (**C**) SCC-7 cells with silenced Mybbp1a levels showed an enhanced migratory phenotype compared to scramble transfected controls (* p-value < 0.03).

One gene that was highly reduced in local recurrences compared to primary tumors encoded the mouse Myb-binding protein 1a (Mybbp1a) (Figure 1). To confirm altered Mybbp1a protein expression we performed immunohistochemical staining on tissue sections derived from the orthotopic mouse tumor model. A strong nuclear staining for Mybbp1a protein was detected in most tumor cells (84%) of primary tumors, which was significantly reduced on tissue sections from local recurrences (35%) (Figure 2). Accordingly, Mybbp1a protein expression was detectable in SCC-7 cells in culture (Figure 3A), which we used for further functional analysis in vitro. We silenced Mybbp1a expression by siRNA technology and assessed the proliferative status by flow cytometry. SCC-7 cells with reduced Mybbp1a levels exhibited impaired proliferation compared to control transfected cells, 12% and 24% cell in S-phase, respectively (Figure 3B), suggesting a positive correlation between Mybbp1a protein levels and tumor cell proliferation in primary tumors. In line with this assumption, staining of tissue sections derived from the mouse model revealed 44% Ki67-positive in the primary tumor, while recurrent tumors with decreased immunoreactivity for Mybbp1a showed a prominent reduction of Ki67 staining (14% of the total cells; Additional file 2: Figure S1).

Next, we analyzed Mybbp1a-siRNA and control transfected SCC-7 cells in a migration chamber and found a significant increase in cell migration upon silencing of Mybbp1a expression (Figure 3C), which was accompanied by reduced E-cadherin protein levels (Figure 3A). In summary, these data demonstrate an opposing role of MYBBP1A in SCC-7 proliferation and migration.

### Expression and function of MYBBP1A in human HNSCC cell lines and tumor samples

**Figure 4:**
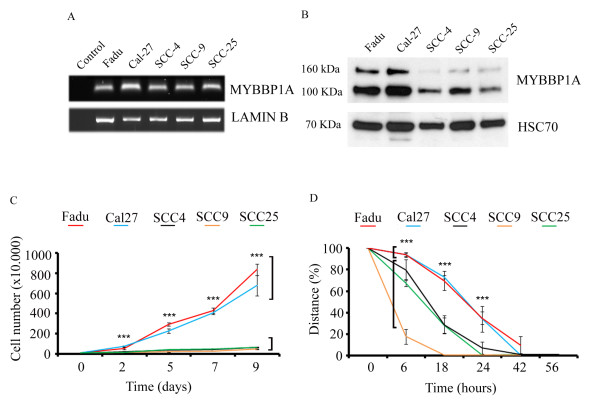
**MYBBP1A expression in human HNSCC cell lines**. (**A**) Comparable levels of MYBBP1A transcripts were detected by semi-quantitative RT-PCR in different human tumor cell lines. LAMIN B transcript levels served as control for cDNA quality and quantity. (**B**) Western blot analysis with nuclear protein extracts revealed strong differences in MYBBP1A protein levels. Detection of HSC70 protein levels served as control for quality and quantity of protein lysates. (**C**) Increase in cell numbers of Fadu, Cal-27, SCC-4, SCC-9 and SCC-25 were counted at the indicated time points and plotted over time (*** p-values < 0.005). (**D**) In vitro wounding scratch assay revealed a significant higher migration rate for SCC-4, SCC-9 and SCC-25 cells compared to Fadu and Cal-27 cells. Migration was determined by measuring the closure of the gap over time and plotted as percentage total of the distance (*** p-values < 0.005).

To further support the opposing role of MYBBP1A in tumor cell physiology, we analyzed its transcript and protein levels in well-established human HNSCC cell lines (Fadu, Cal-27, SCC-4, SCC-9, and SCC-25). Semi-quantitative RT-PCR analysis revealed comparable transcript levels of MYBBP1A in all cell lines (Figure 4A). However, we found a significant difference in protein levels with prominent MYBBP1A expression in Fadu and Cal-27 cells, but reduced protein levels in SCC-4, SCC-9 and SCC-25 cells (Figure 4B), suggesting a post-transcriptional mode of MYBBP1A regulation.

**Figure 5:**
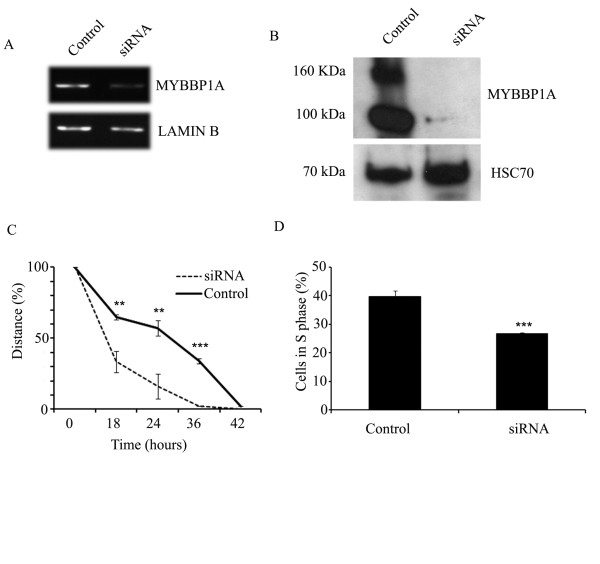
**Opposing function of MYBBP1A expression on cell migration and proliferation in human HNSCC Cal-27**. siRNA-mediated silencing of MYBBP1A expression in Cal-27 cells compared to controls (scramble siRNA) was confirmed by RQ-PCR (**A**) and Western blot analysis (**B**). Lamin B transcript levels served as control for cDNA quality and quantity, while detection of HSC70 protein levels served as control for quality and quantity of protein lysates. Significantly accelerated migration (**C**) and decreased proliferation (**D**) was observed upon silencing of MYBBP1A in Cal-27 cells. Migration was determined as described in Figure 3 and proliferation was quantified by BrdU incorporation and FACS analysis. The graph represents mean values ± SD of the percentage total of cells in S-phase. ** p-values < 0.05, *** p-values < 0.005.

In line with the findings in murine SCC-7 cells, we found a positive correlation between high MYBBP1A protein levels and increased growth rate of human HNSCC cells as shown by quantification of cell growth over time (Figure 4C). In contrast, high MYBBP1A protein levels were negatively correlated with the migration capacity (Figure 4D), further supporting a regulatory function of MYBBP1A as a promoter of tumor cell proliferation and inhibitor of migration. Moreover, SCC-25 cells with low MYBBP1A levels exhibited a significantly higher invasion capacity in a matrigel assay, compared to high expressing cells, such as mouse SCC-7 or human Cal-27 cell lines (Additional file 3: Figure S2). Accordingly, silencing of MYBBP1A expression in Cal-27 cells by siRNA technology (Figure 5A-B) revealed a significant decrease in cell proliferation as determined by BrdU incorporation (Figure 5C) accompanied by increased migration compared to mock controls (Figure 5D).

**Figure 6:**
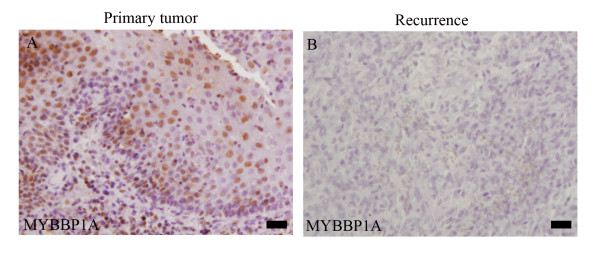
**MYBBP1A protein expression on matched samples of human primary and recurrent tumors**. Representative pictures of immunohistochemical staining for MYBBP1A levels in a primary tumor (**A**) and the respective recurrence are shown (**B**). Scale bars, 50 μm.

Finally, tissue sections of matched pairs of human primary and recurrent tumors were analyzed by immunohistochemistry. In five out of ten HNSCC patients (50%) a prominent and nuclear staining of MYBBP1A protein was easily detected in primary tumors, which was severely reduced in respective recurrent tumors (Figure 6).

## Discussion

In this study, we applied global gene expression profiling on samples of a well-established mouse model of tumor recurrence, and identified differentially expressed candidate genes some of which have been described previously in the context of HNSCC development or tumor recurrence, such as mucins, kallikreins, tryptase alpha/beta 1, claudin-10 and lactotransferrin [15-20]. We also provided experimental evidence for the crucial role of MYBBP1A during malignant progression of HNSCCs.

Originally, MYBBP1A was identified as a c-Myb proto-oncogene product interacting protein [9,10]. Several findings support the assumption that MYBBP1A is a key regulator of tumor cell physiology: (I) in the preclinical mouse model of tumor relapse and in matched pairs of HNSCC patients MYBBP1A showed high expression in primary tumors which was severely reduced in recurrent tumor samples, and (II) MYBBP1A expression in murine SCC-7 cells as well as human HNSCC cell lines support proliferation but was inversely correlated with tumor cell migration.

Hitherto, the molecular mechanism resulting in reduced MYBBP1A protein levels in recurrent tumors remains elusive and will be a major challenge for the future. So far, our knowledge on regulation and function of MYBBP1A protein with regard to physiological and pathophysiological conditions is limited. Concerning post-translational modification proteolytic processing of MYBBP1A has been reported in some cells types [21]. Moreover, MYBBP1A was identified as a novel aurora B kinase substrate, suggesting an important role of MYBBP1A phosphorylation in the regulation of its function [22]. Accordingly, human HNSCC cell lines used in our study exhibited comparable transcript levels while expressing different amounts of MYBBP1A protein, suggesting that MYBBP1A protein levels are at least in part regulated by posttranslational mechanisms. In line with this assumption, a pilot study demonstrates regulation of MYBBP1A protein stability by ubiquitination, since treatment of SCC-25 cells with the proteasome inhibitor MG-132 resulted in an enrichment of the protein when compared to untreated cells (data not shown). Hence, a major challenge for the future will be to unravel the molecular components involved in this process.

Intriguingly, high levels of MYBBP1A protein were associated with increased proliferation but reduced migration and invasion of murine SCC-7 and human HNSCC cell lines, a phenotype that was reverted by silencing of MYBBP1A expression. Recent publications established a critical function for MYBBP1A as an important regulator of distinct transcription factors, such as the proto-oncogenes MYB and NFκB as well as the tumor suppressor p53, implicated in cell cycle control and carcinogenesis [10,11,23,24]. It is worth to note that aberrant regulation of p53 and NFκB is a frequent event in human HNSCC and critically involved in tumor cell proliferation and the malignant phenotype [25,26]. More recently, the molecular mechanism has been elucidated, how mitotic stress and nucleolar disruption activates MYBBP1A resulting in acetylation and accumulation of p53 in a p300-dependent manner [23,24]. The fact that all HNSCC cell lines used in this study express no or only mutated p53 suggests that the physical interaction between MYBBP1A and p53 is not required for its opposing functions on tumor cell proliferation and migration, and it will be interesting to investigate the consequence of MYBBP1A over expression and silencing in primary keratinocytes as well as tumor cells with functional p53.

Although the underlying molecular mechanism, how MYBBP1A regulates the tumor cell physiology remains to be elucidated our data suggest that tumor cells with reduced MYBBP1A protein expression belong to a subpopulation of slow-cycling cells with high mobility. Concerning the later phenotype, improved migration and invasion capacity of tumor cells with low or absent MYBBP1A expression might be, at least in part, due to a reduction in membrane proteins that are critically implicated in cell-cell adhesion, such as E-Cadherin. However, more detailed studies are required to highlight the molecular function of MYBBP1A under physiological and pathological conditions, including cancer. The existence of a slow-cycling subpopulation of MYBBP1A negative tumor cells in HNSCC patients could be of high clinical importance since most current therapeutic regimens target the rapidly proliferating tumor bulk.

## Conclusion

Our data support a model in which MYBBP1A exhibits opposing functions in the pathogenesis of HNSCC. In the early phase of HNSCC development MYBBP1A promotes tumor cell proliferation, while in advanced tumors inactivation of MYBBP1A induces accelerated tumor cell migration and invasion, and thereby hampers the loco-regional control by facilitating lymph node metastasis and tumor recurrence. It will be a major challenge for the future to proof the concept whether the reactivation of MYBBP1A expression could serve as a novel option for anti-cancer therapy for patients with advanced HNSCC and thereby ameliorate the therapy response rate.

## Competing interests

The authors declare that they have no competing interests.

## Authors' contributions

GA carried out the sample preparation for and statistical analysis of the global gene expression profiling study, validation of data, immunostainings of the mouse tumor sections and statistical analysis, human squamous cell carcinoma cell lines characterization, migration and proliferation assay analysis, siRNA knock down, drafted and corrected the manuscript. LF carried out immunohistological staining of tumor sections, its statistical analysis and participated in the migration assay. BG and VM performed proliferation and invasion assays. JK performed immunohistochemical staining of human tumor sections CF pathological inspection of human tumor samples. PP and GD were responsible for human tumor samples organization. PA participated in the design, coordination and the corrections of the manuscript. CS carried out the animal experiments and participated in the corrections of the manuscript. JH was responsible for the experimental design, drafting of the manuscript and conceiving this study. All authors read and approved the final manuscript.

## Additional files

### Probe labeling and illumina sentrix beadchip array hybridization

Biotin-labeled cRNA samples for hybridization on Illumina Mouse Sentrix-6 BeadChip arrays (Illumina, Inc.) were prepared according to Illumina's recommended sample labeling procedure based on the modified Eberwine protocol (Eberwine *et al*., 1992). In brief, 500 ng total RNA was used for complementary DNA (cDNA) synthesis, followed by an amplification/labeling step (*in vitro *transcription) to synthesize biotin-labeled cRNA according to the MessageAmp II aRNA Amplification kit (Ambion, Inc., Austin, TX). Biotin-16-UTP was purchased from Roche Applied Science, Penzberg, Germany. The cRNA was column purified according to TotalPrep RNA Amplification Kit, and eluted in 80 μl of water. Quality of cRNA was controlled using the RNA Nano Chip Assay on an Agilent 2100 Bioanalyzer and spectrophotometrically quantified (NanoDrop).

Hybridization was performed at 58°C, in GEX-HCB buffer (Illumina Inc.) at a concentration of 100 ng/μl cRNA, unsealed in a wet chamber for 20 hours. Spike-in controls for low, medium and highly abundant RNAs were added, as well as mismatch control and biotinylation control oligonucleotides. Microarrays were washed once in High Temp Wash buffer (Illumina Inc.) at 55°C and then twice in E1BC buffer (Illumina Inc.) at room temperature for 5 minutes (in between washed with ethanol at room temperature). After blocking for 5 min in 4 ml of 1% (wt/vol) Blocker Casein in phosphate buffered saline Hammarsten grade (Pierce Biotechnology, Inc., Rockford, IL), array signals were developed by a 10 minutes incubation in 2 ml of 1 μg/ml Cy3-streptavidin (Amersham Biosciences, Buckinghamshire, UK) solution and 1% blocking solution. After a final wash in E1BC, the arrays are dried and scanned.

## Pre-publication history

The pre-publication history for this paper can be accessed here:

http://www.biomedcentral.com/1471-2407/12/72/prepub

## Supplementary Material

Additional file 1 Table S1. Primers used for RQ-PCR. Click here for file

Additional file 2 **Figure S1**. **Proliferation characteristics of primary and recurrent mouse tumors**. (**A**) Ki67 protein expression was analyzed by immunohistochemistry on tumor sections derived from the surgical mouse model used in this study. A prominent nuclear staining was observed both in primary tumors and respective recurrence. (**B**) Quantification of positive nuclei revealed a significant decrease in the amount of Ki67-positive cells in recurrent compared to primary tumors (p-value: 0.0004). Scale bars, 50 μm. Click here for file

Additional file 3 **Figure S2**. **Invasive capacity comparison of SCC-7, Cal-27 and SCC-25**. The invasiveness of the cell lines was assessed in Boyden chambers. SCC-25 shows a higher invasive capacity than the high MYBBP1A expressing cell lines Cal-27 and SCC-7 (p-value: 0.014) (**A**). Click here for file
