# Supplementary material for: Opposing function of MYBBP1A in proliferation and migration of head and neck squamous cell carcinoma cells
Source: BMC Cancer. 2012 Feb 17;12:72. doi: 10.1186/1471-2407-12-72 (PMC3342895; doi:10.1186/1471-2407-12-72)
Supplement: Additional file 1 — Table S1. Primers used for RQ-PCR. [file 1471-2407-12-72-S1.PDF]

Additional files

Probe Labeling and Illumina Sentrix BeadChip array Hybridization

Biotin-labeled cRNA samples for hybridization on Illumina Mouse Sentrix-6 BeadChip arrays (Illumina, Inc.) were prepared according to Illumina's recommended sample labeling procedure based on the modified Eberwine protocol (Eberwine *et al.*, 1992). In brief, 500 ng total RNA was used for complementary DNA (cDNA) synthesis, followed by an amplification/labeling step (*in vitro* transcription) to synthesize biotin-labeled cRNA according to the MessageAmp II aRNA Amplification kit (Ambion, Inc., Austin, TX). Biotin-16-UTP was purchased from Roche Applied Science, Penzberg, Germany. The cRNA was column purified according to TotalPrep RNA Amplification Kit, and eluted in 80 µl of water. Quality of cRNA was controlled using the RNA Nano Chip Assay on an Agilent 2100 Bioanalyzer and spectrophotometrically quantified (NanoDrop).

Hybridization was performed at 58°C, in GEX-HCB buffer (Illumina Inc.) at a concentration of 100 ng/µl cRNA, unsealed in a wet chamber for 20 hours. Spike-in controls for low, medium and highly abundant RNAs were added, as well as mismatch control and biotinylation control oligonucleotides. Microarrays were washed once in High Temp Wash buffer (Illumina Inc.) at 55°C and then twice in E1BC buffer (Illumina Inc.) at room temperature for 5 minutes (in between washed with ethanol at room temperature). After blocking for 5 min in 4 ml of 1% (wt/vol) Blocker Casein in phosphate buffered saline Hammarsten grade (Pierce Biotechnology, Inc., Rockford, IL), array signals were developed by a 10 minutes incubation in 2 ml of 1 µg/ml Cy3-streptavidin (Amersham Biosciences, Buckinghamshire, UK) solution and 1% blocking solution. After a final wash in E1BC, the arrays are dried and scanned.

Supplemental Table 1. Primers used for RQ-PCR

Gene	Forward (5´-3´)	Reverse (5´-3´)
Ovos2	gcggggaaatcgttgcggataatg	gcatggttctgcttgctgatc
Tpasb1	cccaacaaggtcagagtacagc	gtggcggcaggtttacaccatt
Muc10	ggtataagatgtgccctccagg	ggttgcatttgtggttgttgctgg
Dcpp1	ccagaagttggaaaacattcc	ggctgggatcgttagggaagc
Smr3a	ggtcttaggcctctgcattcttg	cattgtagttgcttgtactgtgg
mMybbp1a	gacattgcgaaaccggatcag	gcctgactgaaagagggctag
hMYBBP1A	cgcaccacctgtgccgtgcccggcg	cttggtcaggaaggagctcagtgctg

Supplemental Figure legend S1

Proliferation characteristics of primary and recurrent mouse tumors. (**A**) Ki67 protein expression was analyzed by immunohistochemistry on tumor sections derived from the surgical mouse model used in this study. A prominent nuclear staining was observed both in primary tumors and respective recurrence. (**B**) Quantification of positive nuclei revealed a significant decrease in the amount of Ki67-positive cells in recurrent compared to primary tumors (p-value: 0.0004). Scale bars, 50 µm.

Supplemental Figure legend S2

Invasive capacity comparison of SCC-7, Cal-27 and SCC-25. The invasiveness of the cell lines was assessed in Boyden chambers. SCC-25 shows a higher invasive capacity than the high MYBBP1A expressing cell lines Cal-27 and SCC-7 (p-value: 0.014) (**A**).
