# Supplementary figures and images for: Opposing function of MYBBP1A in proliferation and migration of head and neck squamous cell carcinoma cells
Source: BMC Cancer. 2012 Feb 17;12:72. doi: 10.1186/1471-2407-12-72 (PMC3342895; doi:10.1186/1471-2407-12-72)

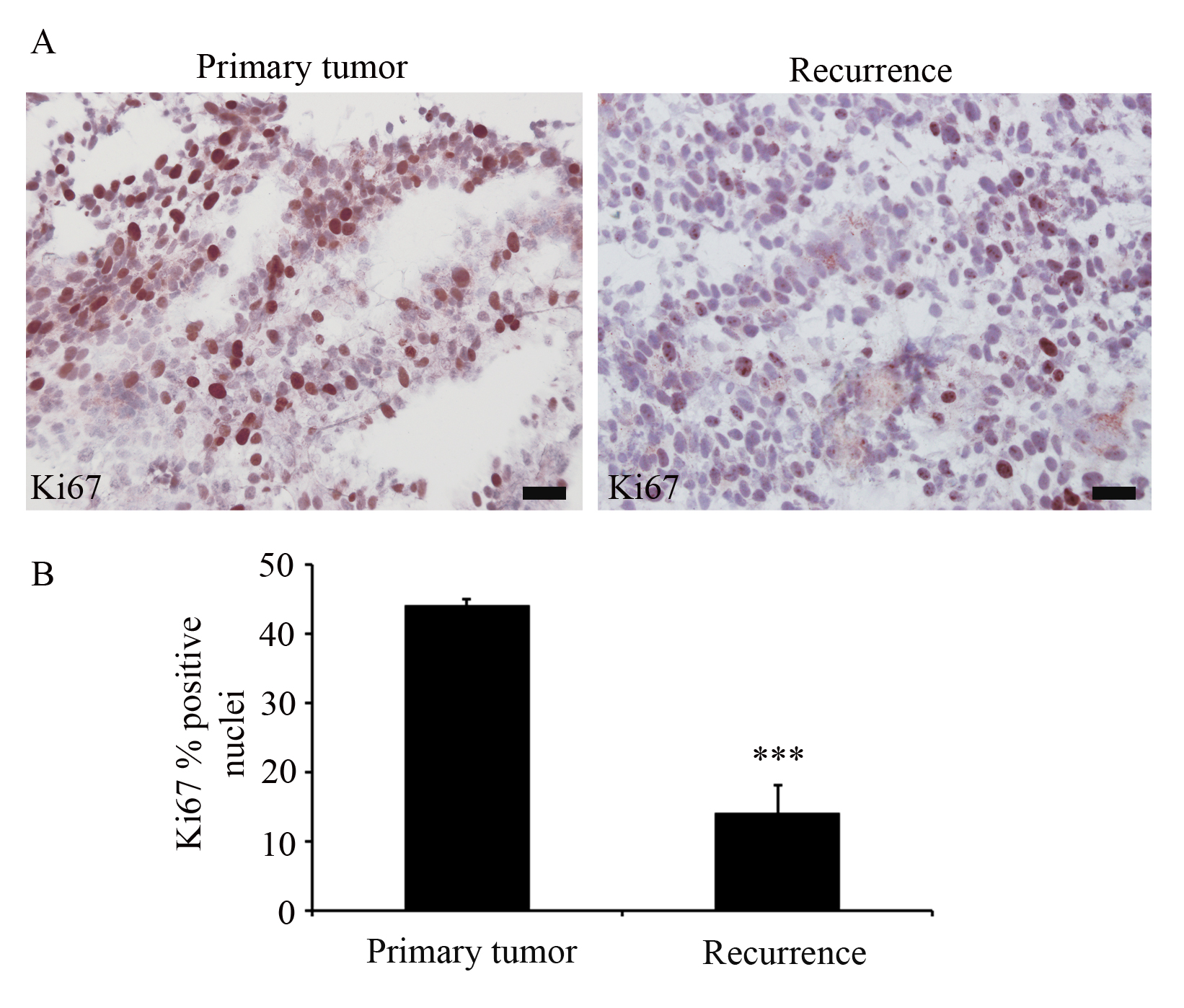

Supplement: Additional file 2 — Figure S1. Proliferation characteristics of primary and recurrent mouse tumors. (A) Ki67 protein expression was analyzed by immunohistochemistry on tumor sections derived from the surgical mouse model used in this study. A prominent nuclear staining was observed both in primary tumors and respective recurrence. (B) Quantification of positive nuclei revealed a significant decrease in the amount of Ki67-positive cells in recurrent compared to primary tumors (p-value: 0.0004). Scale bars, 50 μm. [file 1471-2407-12-72-S2.JPEG]

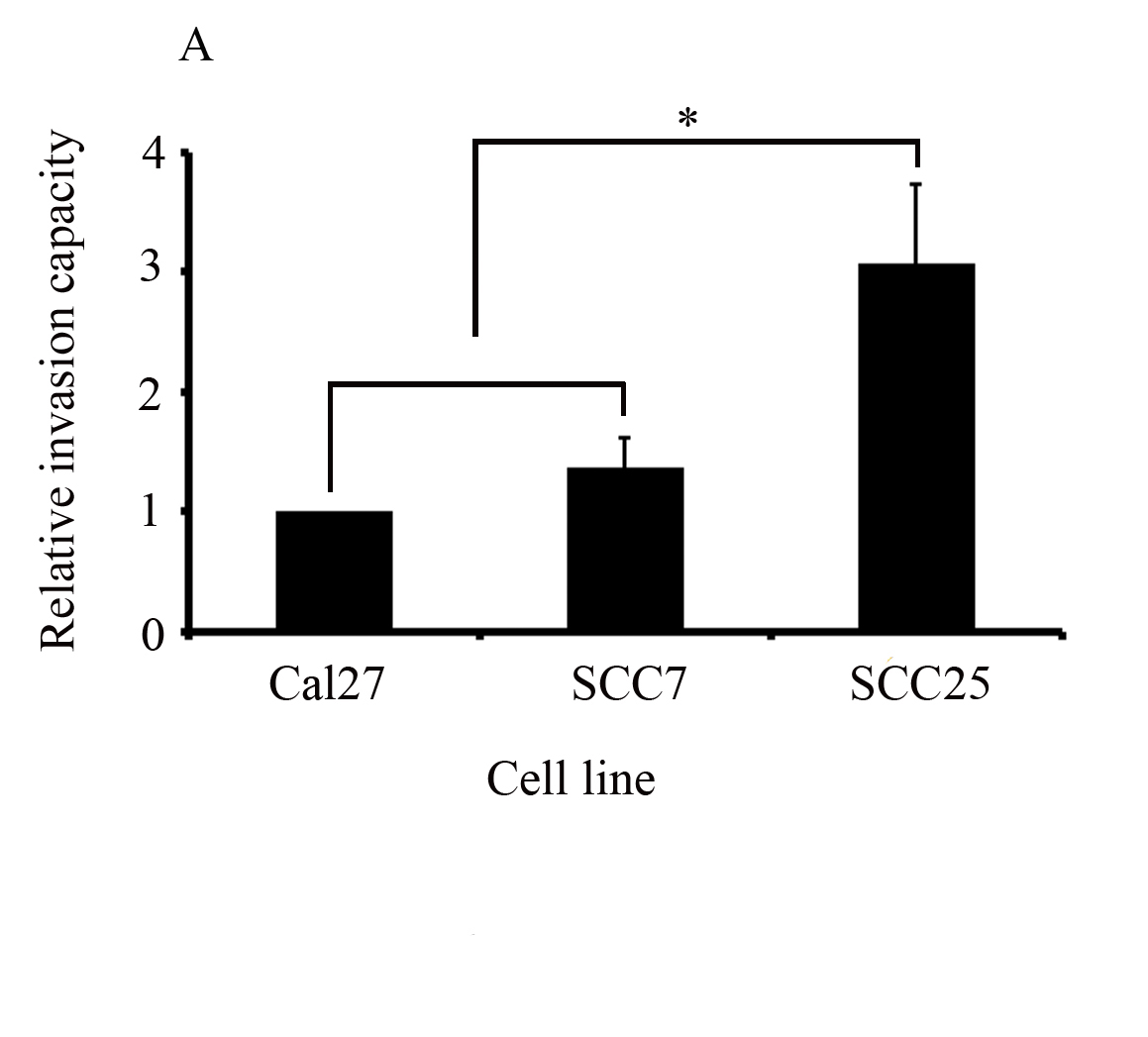

Supplement: Additional file 3 — Figure S2. Invasive capacity comparison of SCC-7, Cal-27 and SCC-25. The invasiveness of the cell lines was assessed in Boyden chambers. SCC-25 shows a higher invasive capacity than the high MYBBP1A expressing cell lines Cal-27 and SCC-7 (p-value: 0.014) (A). [file 1471-2407-12-72-S3.JPEG]
